# Classification of the primary progressive aphasias: principles and review of progress since 2011

**DOI:** 10.1186/s13195-016-0185-y

**Published:** 2016-04-21

**Authors:** Rik Vandenberghe

**Affiliations:** Department of Neurosciences, Laboratory for Cognitive Neurology, KU Leuven, Leuven, Belgium; Neurology Department, University Hospitals Leuven, Leuven, Belgium; Alzheimer Research Centre KU Leuven, Leuven research Institute for Neuroscience & Disease, University of Leuven, Leuven, Belgium

## Abstract

Highly influential recommendations published in 2011 for the classification of the primary progressive aphasias (PPA) distinguished three subtypes: the semantic variant, the nonfluent/agrammatic variant, and the logopenic variant. We review empirical evidence published after 2011 that bears relevance to the validity of the recommended classification scheme. The studies that we review principally rely on monocentric, memory clinic-based consecutive series of PPA patients. We review whether a data-driven analysis of neurolinguistic test scores confirms the subtyping that was based on expert consensus, whether the 2011 subtyping covers the diversity of PPA in a comprehensive manner, and whether the proposed subgroups differ along dimensions that are not explicitly part of the defining criteria, such as diffusion tractography. Data-driven mathematical analyses of neurolinguistic data in PPA broadly confirm the presence of separate clusters corresponding to the subtypes but also leave 15–30 % unclassified. A comprehensive description of PPA requires the addition of the mixed variant as a fourth subtype and needs to leave room for cases fulfilling the criteria for a root diagnosis of PPA but not those of any of the three subtypes. Finally, given the limited predictive value of the clinical phenotype for the underlying neuropathology, biomarkers of the underlying pathology are likely of clinical utility in PPA.

## Background

Consensus recommendations for the classification of primary progressive aphasia were published in 2011 [1], partly motivated by the need to consolidate the logopenic variant (LV) [2, 3] as a third subtype in addition to the nonfluent/agrammatic variant (NFV) [4] and the semantic variant (SV). The latter is also known as semantic dementia [5]. The LV is associated with substantially higher probability of Alzheimer’s disease (AD) compared with the other two subtypes—hence its clinical relevance [2, 3]. We describe the principles of the PPA classification scheme and review evidence that appeared after the recommendations were published and that bears on the validity of this classification scheme and may also point to possible ways in which the classification scheme could be further improved.

## The current recommendations for classifying PPA cases

### Root diagnosis of PPA

The root diagnosis of PPA is based on the objective impairment of language while other cognitive domains (episodic and topographical memory, constructional praxis, etc.) are relatively preserved [6]. In the initial disease stages, impacts on the instrumental activities of daily living are entirely attributable to language problems.

Confusion may arise when the root diagnostic criterion is not fulfilled and the subtyping is applied nevertheless. In that respect it is of particular importance not to confuse PPA or any of its subtypes with the left hemisphere-dominant type of clinically probable AD with prominent language symptoms, a disease entity that has been well-known for a long time [7, 8]. In PPA due to AD, tests of nonverbal domains—for instance, copy of the overlapping pentagons in the Mini Mental State examination or ideomotor praxis—should be, by definition, preserved. On the other hand, in left hemisphere-dominant clinically probable AD, some other cognitive domains besides language are, by definition, also affected and the PPA root criterion is therefore not fulfilled. Some clinical features may be particularly discriminative, such as tests for constructional praxis. Other tests, e.g., of verbal episodic memory, may be less reliable as they will be influenced by the language problems. Neither are nonverbal episodic memory tests particularly useful in this respect in our experience as encoding and retrieval can be affected by the executive dysfunction that can occur in PPA.

Of patients who fulfilled the root criterion of PPA, 40 % had underlying AD [9]. When AD causes PPA, the neuropathology is atypical because of the asymmetric, left hemisphere-dominant distribution of tangles and because of the higher ratio of neocortical-to-entorhinal tangles [10].

### The logopenic variant

**Fig. 1:**
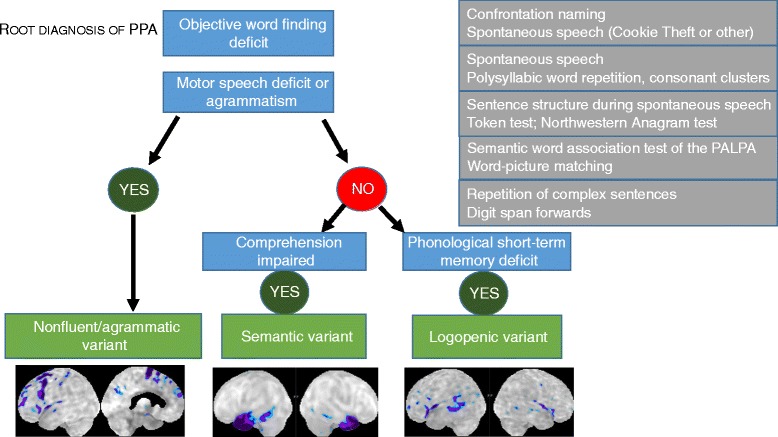
The basic scheme for PPA classification according to the 2011 recommendations. For a more detailed description of positive and negative criteria for assignment to one of the three subtypes we refer to [1]. *Blue*, most critical language dimension; *gray*, some examples of frequently used conventional neuropsychological tests; *green*, subtype. *PALPA* Psycholinguistic Assessment of Language Processing in Aphasia

In PPA LV, spontaneous speech is halting and characterized by fluency disruptions with incomplete words and hesitations [11]. Word finding pauses frequently occur after determiners preceding content words [12]. Grammatical processing and motor speech are relatively preserved (Fig. 1).

Testing repetition is key to the diagnosis of LV. It is critical not to rely on a global repetition score but to take into account the subscores for the different types of materials. In LV, the repetition deficit characteristically affects repetition of longer or complex sentences [13] rather than single polysyllabic words [13, 14]. This differs from NFV with speech apraxia where polysyllabic words will be affected by the motor speech deficit, in particular when consonant clusters are present. One of the least specific and most sensitive tests for repetition concerns the repetition of a string of function words, as in the MiniMental State Examination test. This item can be impaired across the entire spectrum of PPA as well as early in typical AD.

The cognitive deficit underlying the repetition deficit in LV is most likely a short-term phonological memory deficit [3]. In LV, the digit span forward is typically decreased and this deficit does not extend to short-term memory for tones or for location [15]. This clearly differs from SV where digit span forward is often preserved until late into the disease course.

In a hierarchical clustering analysis of 32 cases with PPA excluding semantic variant, cases could be grouped into four clusters [13]. Cluster 1 was characterized by anomia and deficient repetition of sentences and corresponded to LV PPA, cluster 2 to NFV. Cluster 1a was further characterized by phonological paraphasias while cluster 1b lacked these. In cluster 1a all cases were amyloid-positive on positron emission tomography (PET) compared with three out of seven cases in cluster 1b [13]. Across the entire group of non-SV PPA [13], the tests that best discriminated the amyloid-positive from the amyloid-negative cases were the presence of phonological paraphasias and the absence of agrammatism and motor speech disorder. Sentence comprehension and anomia as such did not have discriminative value within this non-SV PPA group [13].

This and other studies suggest that the LV encompasses at least two subtypes [12–14, 16]: one closely resembles left hemisphere-dominant probable AD and the other constitutes a more restricted clinical and anatomical phenotype more similar to the original description [2]. This led Teichmann et al. [12] to propose the term “logopenic aphasia complex”.

In LV, as the disease progresses [16], language impairment may become more widespread [12], leading to problems with single word comprehension [16, 17], single word repetition, syntax production, and verbal memory [14]. Non-language cognitive domains may become affected [14] (for instance, constructional praxis) in such a way that the pattern blends with left hemisphere-dominant clinically probable AD [16]. This clinical course is distinct from that seen in SV where the steady progression typically remains restricted to language, semantic memory, and executive dysfunction [16]. Other patients with LV retain a very circumscribed deficit restricted for years to sentence repetition and word finding difficulties and focal atrophy.

**Fig. 2:**
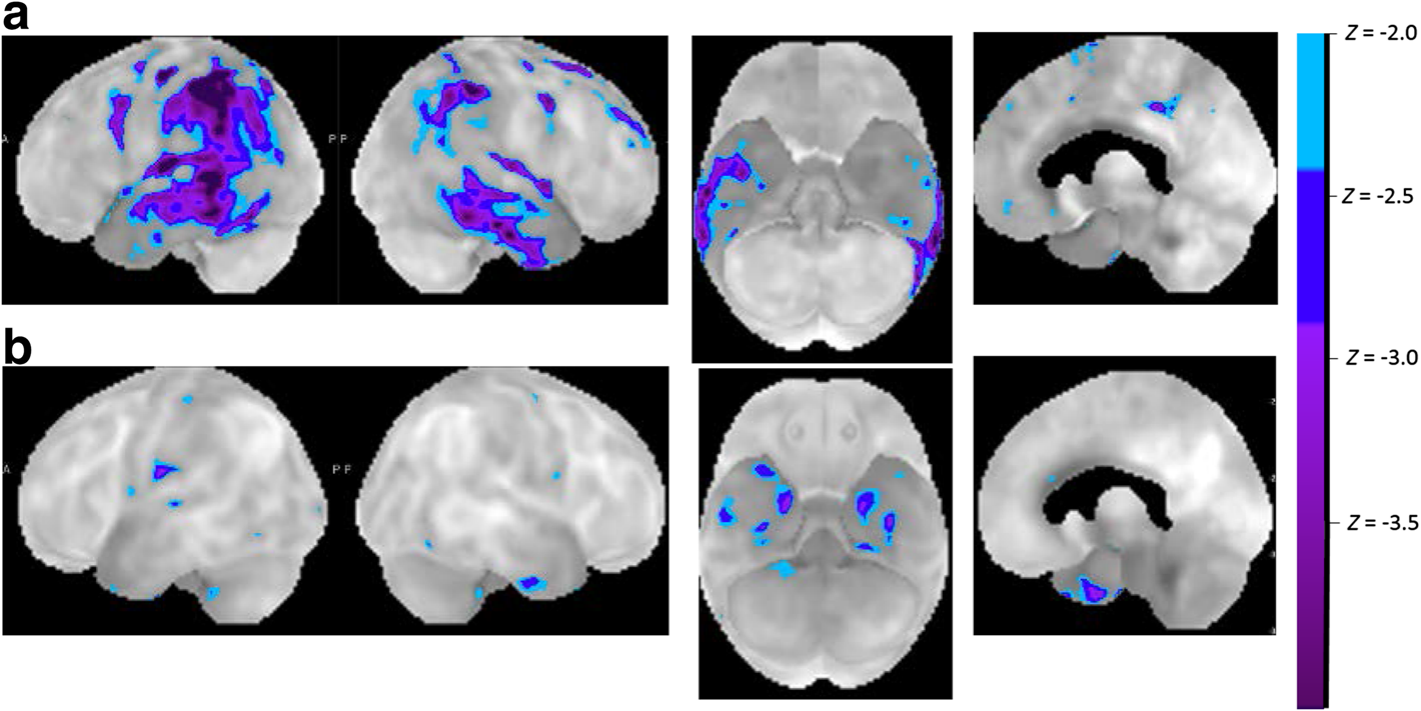
Metabolic patterns in LV. **a** Patient with LV PPA and a pattern characteristic of left hemisphere-dominant Alzheimer’s disease. This would correspond to cluster 1a. **b** Patient with LV PPA and a much more restricted pattern of hypometabolism. This would correspond to cluster 1b. The Z maps are calculated by comparing the individual glucose metabolic pattern to the normal age-matched control database using commercial MIMVISTA software (MIM Software Inc., Cleveland, OH, USA), with a range between 50 and 80 years

In LV cluster 1a [13], the group-based pattern of atrophy, perfusion, and metabolism resembled that seen in the left hemisphere-dominant, clinically probable AD cases [12, 14, 18, 19] (Figs. 1 and 2a). In cluster 1b the damage was more restricted [13] to the temporoparietal transition zone, as originally described by Gorno-Tempini et al. [2, 3] (Fig. 2b). This probably is the key region responsible for the repetition deficit in LV and belongs to the dorsal language pathway [20]. Atrophy in the temporoparietal transition zone correlates with repetition scores and with gesture imitation scores in PPA [21]. In LV, the white matter tracts underneath this cortical region, i.e., the temporoparietal portion of the superior longitudinal fascicle, are also damaged [22].

A second, nearby region that has been implicated in the phonological errors seen in LV is the posterior superior temporal cortex [12, 13]. The posterior superior temporal sulcus is an area of predilection in typical AD from the preclinical [23] to the early clinical stage [24] and activity in this region correlates with word finding scores in clinically probable AD [24] as well as naming latency in preclinical AD [23]. Gray matter density in this region also correlates in LV with scores on lexical decision for words with a meaning that strongly relates to sounds (such as “thunder”) [25].

The positive predictive value of the LV phenotype for a pathological diagnosis of AD is 50–60 % [9, 26, 27], which is lower than what is found in in vivo amyloid biomarker studies in LV (60–90 % [12, 28]). In a meta-analysis of PPA LV series from different centers, the remaining 38 % were due to Tar DNA binding protein 43 (TDP)-associated frontotemporal lobar degeneration (FTLD) [26], with 5–10 % caused by tau-associated FTLD [26]. LV can also be caused by AD combined with Lewy body pathology [9, 27].

### The semantic variant

PPA SV is a distinct disease entity throughout the disease course clinically, anatomically, and neuropathologically—hence the frequently used name “semantic dementia” [5]. Characteristic features apart from anomia are word comprehension deficit and object recognition problems (Fig. 1). In SV, spontaneous speech is fluent, with proportionally fewer nouns and open-content words than in any of the other subtypes and an increase in generic words [11]. As the disease progresses, spontaneous speech becomes restricted to stereotyped utterances consisting of a handful of connected words that may persist for several years and also echolalia. Even in an advanced stage, the clinical neurological examination often remains relatively intact.

Confrontation naming is often severely impaired, and more so than in LV at a comparable stage [6, 9]. Naming errors occur initially, mainly for unfamiliar or atypical items [29], and consist of semantic paraphasias, generalizations, omissions, and circumlocutions. Patients may be able to comprehend the words that they cannot retrieve [30], but as the disease advances they may also not be able to comprehend the word. Object recognition problems and loss of knowledge of visual features of objects further contribute to the confrontation naming deficit in SV. In the written modality, loss of word meaning leads to surface dyslexia [31]. Patients may also experience problems identifying persons (knowledge about individuals) beyond proper name anomia.

The severity of comportmental and personality changes may vary in SV. Both anatomically and clinically, SV can overlap with frontotemporal dementia behavioral variant (FTD bv). The relative preponderance of aphasia versus comportmental disturbances depends on the direction and degree of the left–right asymmetry in the anterior temporal cortex [32].

During stages when word comprehension and object identification are still relatively preserved, SV can resemble LV, but in these circumstances the repetition deficit for specific types of materials, i.e., complex sentences with high working memory demands and the digit span forward, is a discriminative sign in favor of LV. If impaired repetition occurs in an early stage of SV, it is most often restricted to series of function words, a sensitive test for repetition deficit across all three types of PPA.

**Fig. 3:**
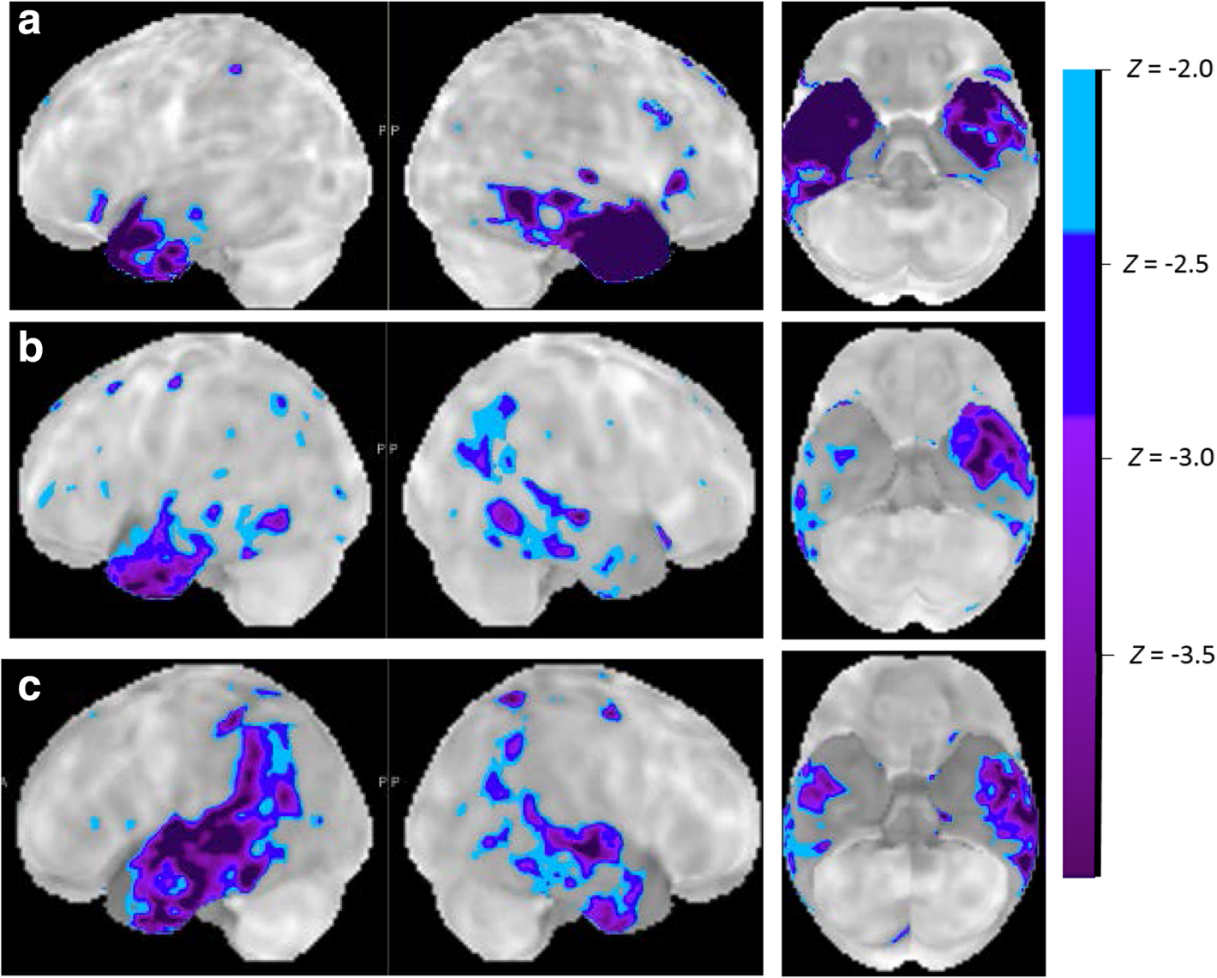
Metabolic patterns. **a** Typical SV PET pattern. **b**, **c** Two PPA cases with a combination of left anterior temporal hypometabolism together with posterior inferior temporal and inferior parietal hypometabolism. This could pose diagnostic problems as there are features of both AD and SV. In the right hemisphere, however, the hypometabolism is more pronounced in posterior than in anterior temporal cortex, suggesting that the damage progressed in the left hemisphere from posterior to anterior temporal cortex. In both cases cerebrospinal fluid AD biomarkers were clearly within the typical AD range (Aβ42 = 393 pg/ml and total tau = 458 pg/ml in **b**; Aβ42 = 525 pg/ml and total tau *>*1200 in **c**)

SV is characterized by a very distinct pattern of anterior temporal atrophy and hypometabolism (Figs. 1 and 3). This may be lateralized to the left or may also affect the right side. Different regions within the anterior temporal cortex may contribute different aspects to the clinical syndrome of SV. Perirhinal cortex belongs to the region of atrophy in semantic dementia [33–35] and scores on tests of semantic memory as well as confrontation naming scores correlate with volume loss in temporopolar cortex and perirhinal cortex [33, 34]. In healthy individuals functional MRI activity patterns in perirhinal cortex, overlapping with the atrophic regions in SV, reflect the semantic similarities between written words [36]. In typical SV, the atrophy may often also impinge on anterior inferior frontal cortex and orbitofrontal cortex [19], which is connected with the anterior temporal pole through the uncinate fascicle. The uncinate fascicle is damaged in SV compared with LV, as well as the inferior longitudinal fascicle [22, 37]. The latter may contribute to functional effects at a distance in the posterior fusiform cortex or the ventral occipitotemporal transition zone. This may account for the visuoperceptual identification problems (structural description system) and the loss of knowledge of visual features that are an integral part of the clinical picture in many SV patients, despite the fact that these regions as such are structurally relatively intact [29, 38].

In rare cases, the anterior temporal damage may be part of a more distributed atrophy and hypometabolism extending contiguously into more posterior temporal cortex and even inferior parietal cortex (Fig. 3b, c). Such a pattern then corresponds to the typical SV anterior temporal region plus the typical AD posterior temporal and inferior parietal region. Distinguishing between SV and left hemisphere-dominant clinically probable AD may require biomarker examination in these circumstances.

In a multicenter series, SV was associated with FTLD-TDP in 69–83 % of cases [26, 39], usually of type C [9]. AD has been described in up to 25 % of PPA SV cases [9, 26], although in most series prevalence of AD in SV is substantially lower (10 %) [40]. In SV due to AD, there is more entorhinal, hippocampal, parahippocampal, and temporal neocortical volume loss compared with non-AD SV [40]. FTLD-tau, principally Pick’s disease, can also occur as a cause [26, 39].

### Nonfluent/agrammatic variant

PPA NFV is characterized by agrammatism and/or speech apraxia (Fig. 1). Agrammatism refers to pathological changes in the morphology of nouns and verbs, word order, and argument structure, with a decrease in mean length of utterance and a decrease in sentence complexity [11]. For a detailed description of the diagnostic features of speech apraxia we refer to Josephs et al. [41, 42]. Schematically, speech apraxia is diagnosed based on abnormal duration of voxels and of inter-segmental intervals (with segments referring to sounds, syllables, or words), unevenness in loudness and pitch and abnormalities in intonational stress, and sound distortions and substitutions, in particular for utterances of increased length and articulatory complexity [41]. This leads to phonetic errors. Phonetic errors must be distinguished from phonological errors which occur in LV in the context of normal prosody and normal sound production and from starting errors that are seen in LV. According to an automated analysis of the speech characteristics in NFV, the changes in relative duration and intensity of vowels as well as the pauses during reading are the most distinctive features [43]. Other qualitative features that may be helpful in the diagnosis of speech apraxia consist of sound and syllable repetitions, groping and effortful speech, speech initiation problems, and abnormalities in coordination with breathing [41].

Most often agrammatism and motor speech deficits occur together (9 out of 25 PPA in Mesulam et al. [6]). More rarely, the motor speech deficit may occur in isolation (1 out of 25 in Mesulam et al. [6]). The latter phenotype has been termed “progressive apraxia of speech” [41]. Since the deficit is limited to motor speech and does not affect language processing strictly speaking, Josephs et al. [41] have argued that this should be set apart from PPA. Inversely, agrammatism can also occur without speech apraxia: a hierarchical clustering analysis in 32 non-SV PPA patients revealed two clusters corresponding to NFV, one in which motor speech disorder co-occurred with agrammatism and one with agrammatism alone [13]. Phenotypically, therefore, one can distinguish three further subtypes within NFV PPA, depending on whether the agrammatism and speech apraxia occur in isolation or together. Over time the exclusivity may disappear [44].

A conspicuous source of heterogeneity within NFV are the associated neurological signs and symptoms that may be present at the initial clinical examination or may appear over the disease course [44]. These signs may point to progressive supranuclear palsy (PSP) or corticobasal degeneration (CBD) as the underlying cause. In 13 patients with progressive apraxia of speech, five evolved into a PSP-like syndrome while in the remaining subjects the speech problems continued to be the most prominent symptom along with progressive extrapyramidal signs [44].

**Fig. 4:**
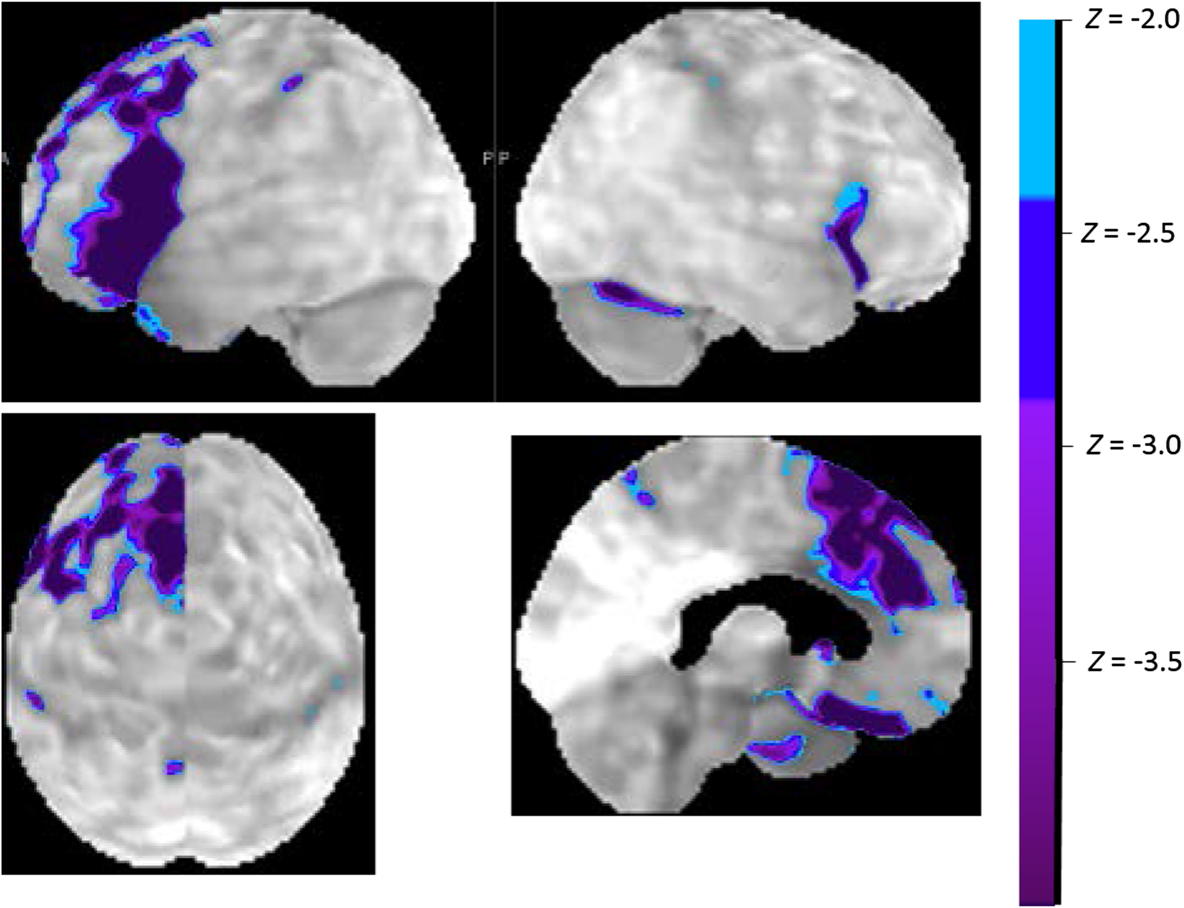
Typical metabolic pattern in NFV showing hypometabolism in inferior frontal, premotor, and supplementary motor cortex

In progressive apraxia of speech, FDG-PET mainly reveals hypometabolism in superior premotor and supplementary motor areas [44, 45] (Fig. 4). Superior premotor involvement correlates with the degree of speech apraxia [45]. As the disease progresses, regions at a distance may become involved, such as inferior parietal or posterior temporal cortex [19]. In the agrammatic variant a more distributed network is involved, including pars orbitalis, triangularis, and opercularis along with superior temporal gyrus and inferior parietal lobule [6, 45]. Therefore, speech apraxia and agrammatism rely on anatomically dissociable mechanisms. Likewise, the white matter tracts involved differ depending on the degree of speech apraxia versus agrammatism [22]: speech production scores mainly correlate with the white matter tract from the inferior frontal gyrus (Brodmann area (BA) 44) and the anterior insula to premotor (BA6 face/mouth area) and supplementary motor cortex (the Aslant tract [46]) as well as connections with putamen and caudate [47]. Sentence comprehension/production, on the other hand, correlates with involvement of the superior longitudinal fascicle and the arcuate fascicle [11, 47]. The dissociation of white matter tract involvement between speech apraxia and agrammatism is not absolute: motor speech scores also show some correlation with integrity of SLF and arcuate [48].

In NFV, 50–70 % of patients are neuropathologically diagnosed as FTLD-tau, corresponding to corticobasal degeneration, progressive supranuclear palsy or Pick’s disease [9, 26, 39]. Exclusive or predominant apraxia of speech predicts a tauopathy rather than a TDP43 proteinopathy [27] and may be more frequently associated with PSP than with CBD [39]. Approximately 20 % of NFV cases are due to FTLD-TDP, usually of type A [27], and 12–25 % to AD [9, 26].

## Cases who do not fit into any of the three classes

In a monocentric series of 84 PPA patients [49], 31 % could not be assigned to any of the three subtypes. In another monocentric retrospective series, this occurred for 5 out of 30 PPA cases [27]. In a third monocentric longitudinal PPA series of 46 patients, 41 % did not fulfill criteria of any of the three subtypes according to Sajjadi et al. [50]. In the latter series the proportion of LV was only 4 % [50], suggesting that the unclassifiables contained cases that in other series could have been assigned to LV. Indeed, in a series by Sajjadi et al. [51] volumetric analysis of 14 unclassified patients revealed an “LV PPA like” pattern. This led the authors to conclude that the unclassifiable cases are more likely “AD-related aphasias.” A case can be unclassifiable because it is missing some of the positive features that are necessary for assigning it to one of the three subtypes proposed by Gorno-Tempini et al. [1]. A case can also be unclassifiable because positive features belonging each to different subtypes occur in combination. The mixed variant is the most typical example of the latter.

### The mixed subtype

**Fig. 5:**
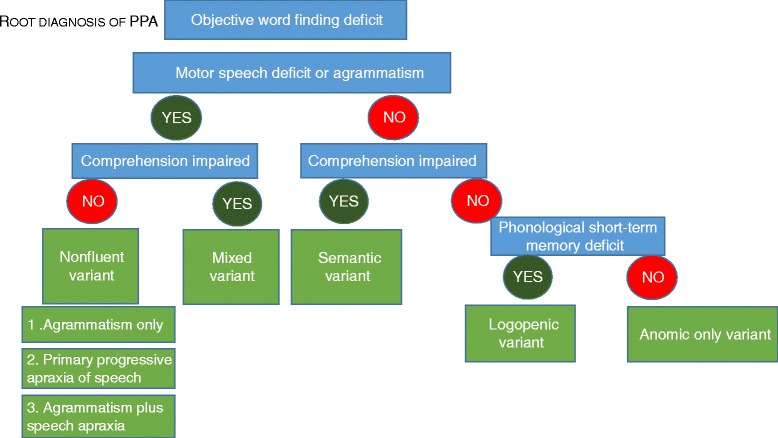
Based on the literature reviewed after the 2011 recommendations, some modifications may be worth considering when describing cohorts for research. Currently the clinical impact of these modifications is insufficiently documented to recommend this updated scheme for use in clinical practice

The mixed subtype exhibits word comprehension deficits along with speech apraxia or agrammatism, a combination of positive findings that does not occur in the current classification [6, 52] (Fig. 5). In our experience, this mixed variant is not rare. In a consecutive series of PPA cases at our memory clinic, 3 out of 21 are of the mixed variant, a proportion similar to that reported by Mesulam et al. [6] (2 out of 25). A group-based volumetric analysis of mixed PPA revealed atrophy in inferior frontal, superior temporal, and anterior temporal cortex [6]. Among six patients with mixed variant PPA, four had AD as underlying pathology, one had FTLD-tau, and one had mixed pathology of AD and TDP-A [9].

### Anomic-only PPA

Patients may fulfill the root criterion of PPA based on their anomia, in the absence of any of the other features that distinguish between subtypes, most notably normal performance on repetition of complex sentences and on word comprehension and no motor speech problems that would permit assignment to one of the three subtypes (Fig. 5). The prevalence in a series of PPA patients was 3 out of 25 [6]. A somewhat related subclass are PPA-L*, who have prominent word finding problems during spontaneous speech but have relatively preserved confrontation naming. PPA-L* have a hesitant spontaneous speech but are missing the repetition deficit typical of LV [9]. It is conceivable that these cases may develop some of the distinguishing features later in the course of the disease.

## Towards an etiological diagnosis of PPA

The clinical utility of biomarkers such as cerebrospinal fluid Aβ42 and amyloid PET in patients with PPA presenting in a memory clinic depends on the difference between pre- and post-test odds of an etiological diagnosis [53] and on the clinical benefit of potential changes in patient management.

### Increase in diagnostic accuracy

None of the three phenotypic subtypes entirely excludes the possibility of AD as the underlying cause. PPA LV has the highest positive predictive value for AD neuropathology but, even so, neuropathological series show a prevalence of AD pathology in PPA LV of only 50–60 % [9, 26]. Inversely, PPA SV or NFV are far more often due to FTLD but can be caused by AD in 10–25 % of cases [9, 26]. The prevalence of AD as the underlying cause in the other categories, such as mixed or anomic variant, is currently unknown. Hence, the highest yield of biomarkers would probably be obtained in LV, anomic or mixed-variant PPA, and in SV in an early phase.

### Clinical utility

The clinical utility of an etiological diagnosis in PPA has not been proven empirically. Conceivably, patients with PPA due to AD may have a cholinergic deficit and hence benefit from cholinesterase inhibitors. Prognostically, the time course expected in patients with PPA due to AD may differ from that in PPA due to a TDP43 proteinopathy or a tauopathy such as CBD or PSP. For instance, the behavioral manifestations may be qualitatively different in FTLD compared with AD, with more obsessive-compulsive behaviors, lack of empathy, and eating abnormalities. In case of PPA due to tauopathy, limb apraxia, gait and postural problems, and dysphagia may impair the autonomy of the patient earlier in the course of the disease compared with PPA due to AD. Inversely, the cognitive domains affected over the disease course in LV due to AD may expand into nonverbal domains. A correct etiological diagnosis at the initial stage may help the patient, caregiver, and physician to anticipate specific problems which may benefit patient management. A correct etiological diagnosis in vivo could also be a prerequisite for success in clinical drug development aimed at disease modification.

## Need for modification of the criteria?

### Unclassified PPA

In itself the observation that a substantial proportion of cases who fulfill the root criterion of PPA do not fall into one of the subtypes (the anomic-only variant) is not necessarily a reason for a revision, as long as these cases are not “forced” into one of the subtypes when communicating research findings or in clinical practice. It is important to recognize in the clinic and in research that the three subtypes do not encompass the full spectrum of PPA phenotypes, as described above [27]. The question then becomes whether within the unclassifiable cases some patterns can be discerned that merit the status of an additional subtype (Fig. 5).

### Addition of subtypes

For the purpose of communicating research findings we believe that there is sufficient evidence and also a need to allow for one additional denotation: the mixed subtype. Based on the prevalence reported, ignoring this subtype would lead to misclassification of about 15 % of cases in research papers on PPA. This additional subtype constitutes an important target group for further research to determine the anatomical and etiological basis and the potential relevance for patient treatment.

### Subdivision of subtypes

Within the NFV, a more fine-grained level of description would specify the agrammatic-only variant, the speech apraxia-only variant, and the combination of both [42]. There are two caveats: it remains to be proven that the underlying neuropathology differs sufficiently between these subdivisions; and such a subdivision heavily relies on evidence that either motor speech or grammatical processing is preserved and this critically depends on the sensitivity of tests and the disease stage.

Within the LV it may be more accurate to distinguish between a subgroup with more widespread language involvement and broader anatomical involvement resembling left hemisphere-dominant AD and a subgroup with LV restricted to phonological working memory deficit and focal damage of the temporoparietal transition zone. The more restricted form has a lower likelihood of AD than the more widespread form.

## Conclusions

Obviously, from the patient and caregiver perspective, a revision would be justified if it has a positive impact on the management of individual patients, as was the case, e.g., for the LV subtype as a recognition of AD as the frequent underlying cause. The 2011 recommendations had an impact beyond research. Before a modification would be deemed of use for clinical practice, more empirical data would be needed regarding the impact of these modifications on prediction of the temporal course or neuropathology and on patient management. Clinically, in our opinion, a higher benefit will be gained from implementing in vivo biomarkers of underlying neuropathological lesions, such as cerebrospinal fluid biomarkers for AD or amyloid PET [53, 54], than from ever-increasing sophistication at the clinical-phenotypical level.

## Abbreviations

AD: Alzheimer’s disease
BA: Brodmann area
CBD: corticobasal degeneration
FTLD: frontotemporal lobar degeneration
LV: logopenic variant
NFV: nonfluent/agrammatic variant
PET: positron emission tomography
PPA: primary progressive aphasia
PSP: progressive supranuclear palsy
SV: semantic variant
TDP: Tar DNA binding protein 43
